# Intraoperative Hypotension in a Patient with Antithrombin Deficiency, Bilateral Pulmonary Emboli, and Cefazolin Allergy

**DOI:** 10.7759/cureus.13653

**Published:** 2021-03-02

**Authors:** Aaron R Muncey, Nasrin N Aldawoodi, Ahish Chitneni, Jamie P Hoffman, Allan R Escher

**Affiliations:** 1 Anesthesiology, Moffitt Cancer Center, Tampa, USA; 2 Anesthesiology, H Lee Moffitt Cancer Center and Research Institute, Tampa, USA; 3 Physical Medicine and Rehabilitation, AT Still University, Arizona, USA; 4 Anesthesiology/Pain Medicine, H Lee Moffitt Cancer Center and Research Institute, Tampa, USA

**Keywords:** antithrombin iii, pulmonary emboli, follicular neoplasm, allergy and anaphylaxis, thyroid neoplasm, antithrombin deficiency, hypercoagulable state, mottling, tryptase, stanford emergency manual

## Abstract

In medicine, the search for a clear answer can at times be elusive. However, this does not necessarily preclude the administration of intelligent and thoughtful therapeutic treatments. Here, we describe a complicated emergent event of severe hypotension and near-arrest that occurred in the operating room in a young, healthy woman undergoing outpatient thyroid surgery. We detail the situation as it presented in the operating room and the measures taken to rule out potential life-threatening diagnoses and develop a thoughtful treatment plan. We further describe the evidence for and against the two remaining diagnostic possibilities: anaphylaxis versus acute pulmonary embolism.

## Introduction

Emergent situations are inherent to the profession of anesthesiology. The case report described here involves a patient who had occult antithrombin (AT) deficiency, diagnosed in the aftermath of an emergent near-arrest that occurred after anesthesia induction and prior to surgical incision in a healthy young woman presenting for outpatient resection of a thyroid neoplasm. We describe the clinical features encountered and steps taken during this complicated emergent event, which exhibited signs of both acute pulmonary embolus (PE) and anaphylaxis. We further discuss the differential diagnosis and how diagnoses were quickly ruled out to best optimize treatment.

AT is an endogenous anticoagulant produced by the liver that inhibits activated clotting factors in plasma, most notably thrombin (factor IIa) and factor Xa. AT causes less significant inhibition of factors IXa, XIa, and XIIa. AT also inhibits several serine proteases such as plasmin, kallikrein, urokinase, and tissue plasminogen activator [1].

AT deficiency has a reported prevalence of one in 500 to one in 5,000 in the overall population. It is transmitted genetically in an autosomal dominant manner, and patients living with the disease tend to be heterozygotes, as the extremely rare cases of homozygous AT deficiency result in death in utero [1]. Due to the deficiency, patients typically present in a hypercoagulable state with recurrent venous thromboses that incur elevated mortality risk secondary to pulmonary emboli [1-2].

Two different types of AT deficiencies exist in the patient population. Type I AT deficiency (12% of AT-deficient patients) results in decreased quantity and function of antithrombin and type II AT deficiency (88%) results in normal quantity and abnormal function. Type II AT deficiency is further subdivided into three different forms: IIa in which the defect is in the thrombin binding domain, IIb in which the defect is in the heparin-binding domain, and IIc, the mixed or pleiotropic form [1].

Perioperative anaphylaxis occurs at an estimated frequency of one in 1,250 to one in 18,600 cases [3]. This acute event can pose a true life-threatening emergency, and mortality is estimated at 3% to 9% [4]. Early detection and treatment are key to patient survival and prevention of associated complications [5- 6]. While any perioperative medication or material can pose an anaphylactic risk, the most common reactions are to the neuromuscular blocking agents, antibiotics, disinfectants, and latex [6].

## Case presentation

A 36-year-old female with a past medical history of prediabetes and mildly elevated body mass index (BMI) of 28 kg/m^2^ presented to the outpatient surgery center for resection of a right thyroid nodule consistent with follicular neoplasm (Hurthle Cell Type, Bethesda IV) with NRAS and copy number alterations. She had no known medication allergies and no significant history of smoking, alcohol, or drug use. She was not taking any home medications, only a daily multivitamin. Family history revealed a brother with a prior transient ischemic attack and coronary artery disease in both her father and brother.

The patient was given 2 mg of intravenous (IV) midazolam and taken to the operating room (OR) for induction of anesthesia. After a standard World Health Organization (WHO) timeout, anesthesia was induced at 9:13 am with 150 mg of IV propofol preceded one minute prior by 50 mcg of IV fentanyl and 60 mg of IV lidocaine. After induction, she received 100 mg of IV succinylcholine for muscle paralysis prior to intubation, which was uneventful. At 9:15 am, she received 10 mg IV dexamethasone for postoperative nausea and vomiting (PONV) prophylaxis. The next medication administered was 2 gm of IV cefazolin at 9:20 am.

At this time the patient’s pulse rate was modestly increased at 108 beats per minute (bpm) compared with her baseline of 71 bpm. Pulse oximetry (SpO2) measured 100% on a fraction of inspired oxygen (FiO2) of 67%. Her blood pressure was stable and end-tidal carbon dioxide (EtCO2) measured 34 cmH2O. By 9:23 am, three minutes later, she developed sinus tachycardia in the 120s, which increased to the 140s by 9:25 am despite an additional dose of 50 mcg of IV fentanyl and 10 mg of IV esmolol. Blood pressure was still stable with a mean arterial pressure (MAP) of 101 mmHg. Within five minutes, the EtCO2 decreased precipitously to 16 cmH2O, whereupon the noninvasive blood pressure readings became unmeasurable. Tachycardia persisted, and the patient suffered from severe hypotension despite multiple boluses of phenylephrine (0.1 mg), epinephrine (0.1 mg, 0.4 mg, and 0.5 mg), and vasopressin (2 units to 5 units). The patient had clear lungs on auscultation and no significant increase in peak inspiratory pressures as measured by the ventilator. A violaceous mottling was noted in the hands and on the chest.

The operation was canceled, and a rapid response team alert implemented to enlist additional support. The FiO2 was increased to 100%, and the volatile anesthetic vaporizer turned off. The surgeon came to the head of the operating table to manually palpate a carotid pulse, which was maintained despite the inability of the automated cuff to detect blood pressure. Palpation of the carotid pulse continued until the anesthesia team successfully placed a brachial arterial line via ultrasound. A femoral central line was placed emergently by the surgical team. The patient’s vital signs finally stabilized on a titrated epinephrine infusion. An arterial blood gas revealed acute metabolic acidosis; the patient received two ampoules of 8.4% sodium bicarbonate and an ampoule of 10% calcium chloride. Diphenhydramine and famotidine were given presumptively for anaphylaxis, though the differential also included acute PE given her history of malignancy. 

**Figure 1:**
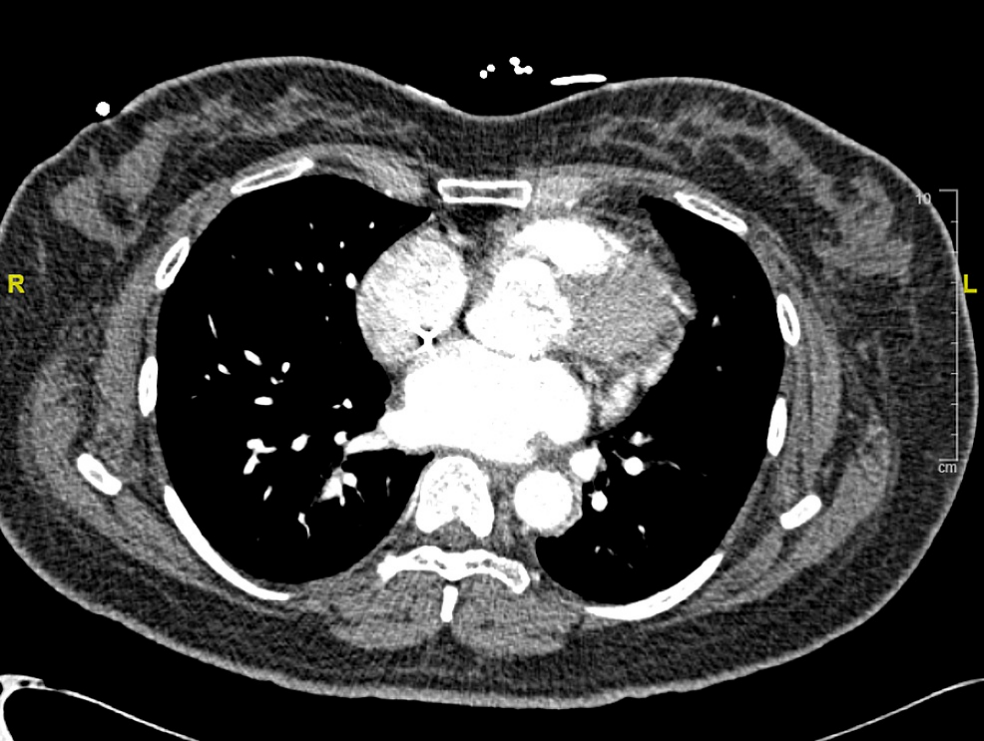
CTA of the chest performed on POD 1 revealed subtle, small peripheral pulmonary arterial filling defects noted in the right upper and left lower lobes consistent with small pulmonary emboli. Enlargement of the main pulmonary artery, which may be seen in the setting of pulmonary arterial hypertension. CTA, computed tomography angiogram; POD, postoperative day

A serum tryptase sent several hours after the initial critical event was found to be elevated at 14.4 ug/L. The following day, a computed tomography angiogram (CTA) of the chest revealed subtle small peripheral pulmonary arterial filling defects noted in the right upper and left lower lobes consistent with small pulmonary emboli and enlargement of the main pulmonary artery. Small peripheral pulmonary arterial filling defects were noted in the right upper and left lower lobes consistent with small pulmonary emboli and enlargement of the main pulmonary artery, which may be seen in the setting of pulmonary arterial hypertension (Figure 1).

**Figure 2:**
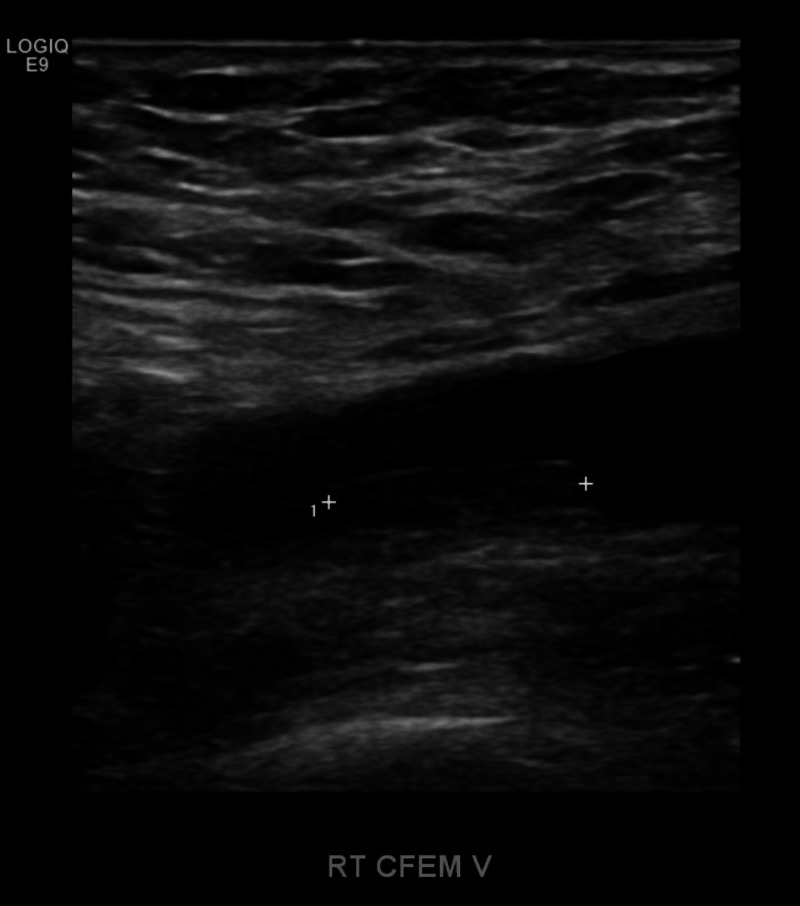
US of the lower extremities revealed a chronic DVT in the right common femoral vein as indicated between the hash marks US, ultrasound; DVT, deep venous thrombosis

**Figure 3:**
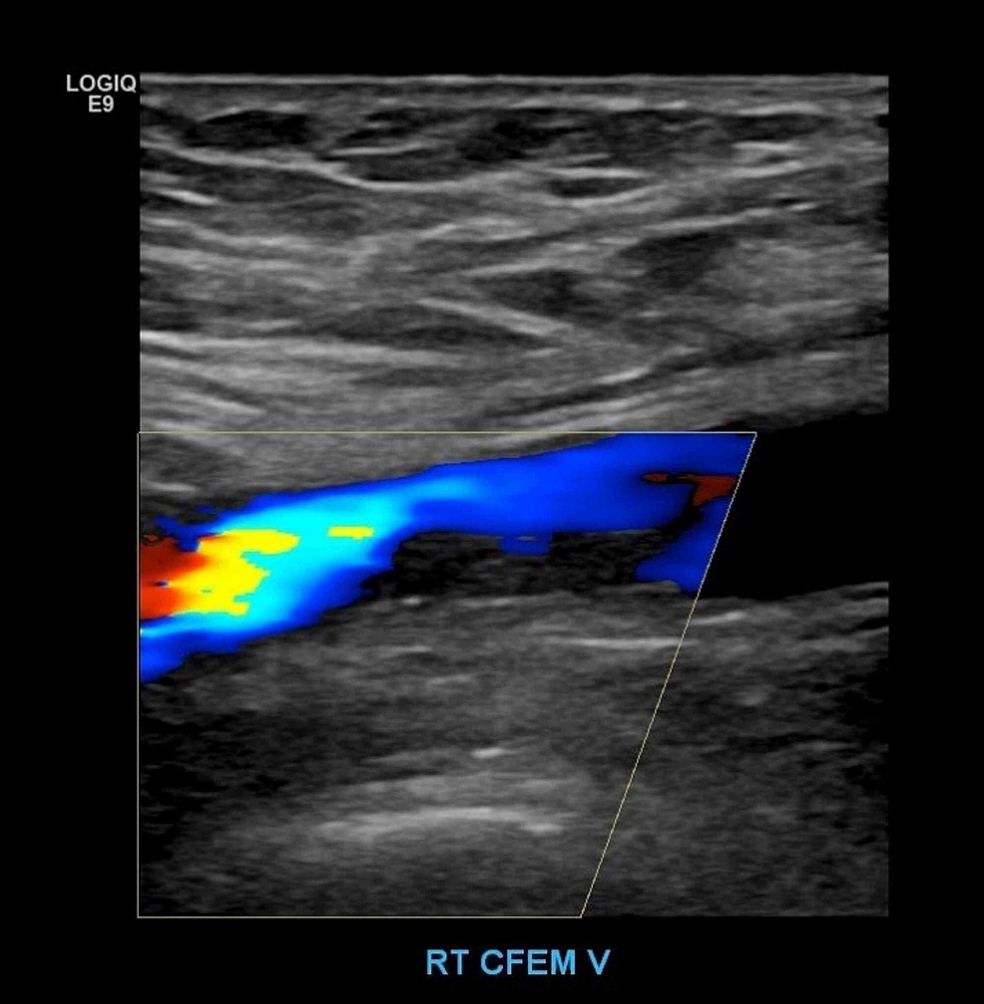
Doppler color flow demonstrated the right common femoral DVT was nonocclusive. DVT, deep venous thrombosis

Lower extremity Doppler imaging followed, revealing a mild eccentric nonocclusive right common femoral vein thrombus that appeared chronic (Figures 2-3).

**Figure 4:**
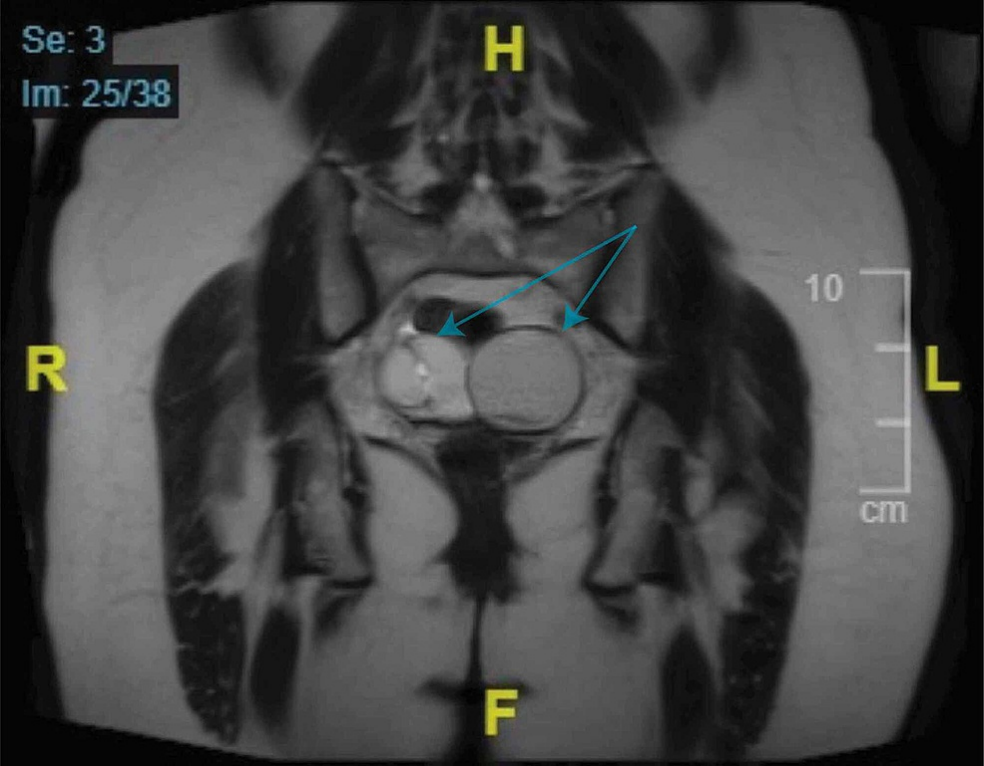
Coronal MR image of the pelvis showing bilateral adnexal cystic masses MR, magnetic resonance

Doppler of the right upper extremity revealed an incidental finding of an occlusive thrombus in the distal basilic vein. The patient was started on full-dose enoxaparin (80 mg, subcutaneous, every 12 hours) and a later workup for inherited coagulopathies ensued. The patient was found to have decreased AT activity measuring 65% (normal range: 76-128%) and decreased AT antigen of 54% (normal range: 82-136%). These two features are indicative of type I AT deficiency [1]. Further imaging suggested that ovarian masses resulting in venous compression could have contributed to the chronic deep venous thrombosis (DVT) and subsequent PE (Figure 4).

The patient was extubated on postoperative day (POD) 1 and discharged home on POD 3. A transesophageal echocardiogram was not performed. After discharge, she had a follow-up with both hematology and allergy/immunology. At her hematology appointment, she was transitioned from enoxaparin to apixaban, which she will remain on indefinitely with enoxaparin bridging, as needed, for surgical procedures. Testing by her allergist revealed a high degree of reactivity to cefazolin, who suggested this as the possible cause of her near-arrest but noted: “it is also possible that another medication or medical event was responsible for the reaction in the OR and not due to cefazolin.” Of note, the patient was started on empiric antibiotics the first day into her ICU course postoperatively because of elevated lactic acid. At that time, she received two doses of ceftriaxone, another cephalosporin, for treatment without incident. She has had several surgeries since, in which she received the same anesthetic agents and medications including midazolam, fentanyl, lidocaine, propofol, succinylcholine, and dexamethasone, all without incident.

## Discussion

This case, in which an unexpected near-arrest occurred intraoperatively, demonstrates the importance of maintaining vigilance during anesthetic administration in the operating room, focusing not only on the vitals and monitors but also on the patient who is often obscured or partially accessible due to surgical drapes. The key event noted here, which heralded the subsequent differential diagnosis, was a constellation of changes in the patient’s vital signs and physical exam noticed by the anesthesiologist. These included a sudden drop in end-tidal CO_2_ and O_2_ saturation, accompanied by severe hypotension and tachycardia.

According to the Stanford Emergency Manual, there are several life-threatening conditions that can present intraoperatively in this manner, including PE, anaphylaxis, myocardial infarction (MI), pneumothorax, anesthetic overdose, hemorrhage, and aspiration [7]. In this case, these diagnoses were considered and ruled out until only two realistic possibilities remained: pulmonary embolism versus anaphylaxis. An MI was extremely unlikely in a young healthy woman with no significant cardiac risk factors. Pneumothorax was unlikely given the absence of increased peak airway pressures on the ventilator nor a precipitating event to cause such a complication. The patient had typical doses of anesthetic for her age and weight and was at less than one minimum alveolar concentration (MAC) of sevoflurane anesthesia, an end-tidal concentration of 1.69%, when the event occurred, thus ruling out anesthetic overdose. A hemorrhage was ruled out by the timing since surgery had not commenced. And aspiration was unlikely given that the patient was appropriately nil per os (NPO) for her elective surgery with no risk factors such as gastroesophageal reflux (GERD) or gastroparesis, nor was any evidence of aspiration witnessed during intubation.

There was compelling evidence to support the diagnosis of both pulmonary embolism and anaphylaxis in this rapidly evolving emergent situation. Acute pulmonary emboli are known to cause a sudden increase in dead space and a concurrent drop in EtCO2 with hypoxia and tachycardia [8]. Violaceous mottling was noted in the fingertips, which is more likely to be seen in acute pulmonary embolism versus anaphylaxis where vasodilation and hyper-perfusion occur. A diagnosis of anaphylaxis was possible given the timing of the event, just shortly after anesthetic induction and antibiotic administration, in which a host of medications were given in a short period of time [6]. Of note, there were no increased peak airway pressures or evidence of bronchospasm, which is typically associated with anaphylaxis.

Fortunately, both conditions can be treated similarly, and the strategic choice of epinephrine as the vasopressor, in this case, allowed for supportive treatment of suspected anaphylaxis while also covering the possibility of acute pulmonary embolus. Epinephrine is the first-line treatment for severe anaphylaxis because it decreases mediator release from mast cells, reverses the associated cardiovascular collapse that occurs secondary to increased vascular permeability, and reverses airflow obstruction and bronchospasm [5,9-10]. Early administration is key, as delays result in increased mortality [5-6]. In this case, boluses of epinephrine were given shortly after the initial event. For severe pulmonary embolism with hemodynamic instability, epinephrine is also considered a first-line agent (in addition to norepinephrine and isoproterenol). These medications, which increase contractility and are positive dromotropes, help mitigate the right ventricular overload and hemodynamic compromise, which can occur with a significant PE [8]. Interestingly, in this case, the patient’s CTA noted enlargement of the pulmonary artery and evidence of pulmonary hypertension. This indicates that the significant burden of her multiple pulmonary emboli may have contributed to the event.

This case was unique in that even after the workup that followed the critical event, it remains unclear whether the patient’s near-arrest occurred secondary to anaphylaxis or pulmonary emboli. The patient’s elevated tryptase of 14.4 ug/L was evidence of anaphylaxis. Tryptase, a serine protease released by mast cells, becomes elevated during acute anaphylaxis. While many centers consider the normal range for tryptase to be less than 11.4 ug/L, based on the manufacturer ThermoFisher (Waltham, Massachusetts), there is variability in this reference range, with some thresholds as low as 8.23 ug/L and others as high as 14 ug/L [11]. A recent study on intraoperative anaphylaxis found a threshold of 15.7 ug/L to be highly predictive of intraoperative anaphylaxis [12]. A 2010 consensus equation was developed by an international working group relating tryptase levels to baseline, where the upper limit for anaphylaxis would be considered > 1.2x baseline tryptase + 2 ug/L [11]. Unfortunately, in this case, we did not have a baseline for comparison. The value of 14.4 ug/L may or may not indicate significant elevation depending on the patient’s baseline, and the basal serum tryptase level is < 15 ug/L in 95% of the population [13]. The patient did have allergy testing, which confirmed a high degree of reactivity to cefazolin and she has since tolerated all other potential triggering medications without incident. The timing of cefazolin administration was consistent with the diagnose of anaphylaxis. Our search of the literature did not reveal a case report depicting both anaphylaxis and pulmonary embolus at the same time.

The patient was also diagnosed with multiple pulmonary emboli, a right lower extremity DVT, and a right upper extremity superficial venous thrombosis in the immediate workup that followed her near-arrest. She also had decreased AT activity and AT antigen levels indicative of type I AT deficiency. Given this newly diagnosed coagulopathy and evidence of pulmonary artery enlargement and pulmonary hypertension on CTA, it is certainly possible that acute pulmonary emboli could have caused the hypotension and near-arrest. It is also possible that acute anaphylaxis in the setting of pre-existing pulmonary emboli may be the true explanation of the patient’s near-arrest.

## Conclusions

This complicated case of an intraoperative near-arrest in an otherwise healthy young woman during elective surgery underscores the importance of the differential diagnosis when critical events occur in the operating room. While we may never know the true cause of this untoward intraoperative event, suspected anaphylaxis versus acute pulmonary emboli, the astute anesthesiologist was able to rule out the least likely causes and provide treatment, with a clear benefit for both pathologic states. In medicine, the search for a clear diagnosis and answer can, at times, be convoluted or even impossible. This is the space in which experience, quick thinking, and a basic understanding of all potential possibilities can be life-saving, as was the case with this patient.
